# Supplementary material for: Improving citizen-government interactions with generative artificial intelligence: Novel human-computer interaction strategies for policy understanding through large language models
Source: PLoS One. 2024 Dec 17;19(12):e0311410. doi: 10.1371/journal.pone.0311410 (PMC11651614; doi:10.1371/journal.pone.0311410)
Supplement: Attachment — Submitted filename: Response to Reviewers.pdf [file pone.0311410.s001.pdf]

Dear Editor and Reviewers,

We sincerely thank you for your thoughtful and constructive feedback on our manuscript. We have carefully addressed all the comments and suggestions, resulting in significant improvements to our paper. Below, we provide a point-by-point response to each reviewer's comments, showing that our revised paper has fully addressed these concerns.

Reviewer #1:

1. Present the novelty of the paper in the abstract.

   Response: We have enhanced the abstract to clearly articulate the novelty of our approach. Specifically, we added: "This novel integration of RAG and LLMs for policy communication represents a significant advancement over traditional methods, offering unprecedented accuracy and accessibility." This addition emphasizes the innovative nature of our work within the context of government-citizen communication.

2. Improve the Image Quality.

   Response: We have fully examined the image quality, and we have not found particular flaws or problems. Please click the external link to view the image since the smaller image might seem fuzzy. Please let us know if there are any problems we need to address before we move forward.

3. Add some results in the abstract.

   Response: We have incorporated key results into the abstract, stating: "Our system demonstrated high accuracy, averaging 85.58% for Chinese and 90.67% for US policies." This addition provides concrete evidence of our system's effectiveness.

4. Add the fullform of AI in the title.

   Response: We have updated the title to include the full form of AI: "Improving Citizen-Government Interactions with Generative Artificial Intelligence: Novel Human-Computer Interaction strategies for Policy Understanding through Large Language Models."

5. In the last paragraph of introduction add the structure of the paper.

   Response: We have added a paragraph at the end of the introduction (lines 80-89) outlining the structure of the paper, providing a clear roadmap for readers.

6. Related work section is missing.

   Response: We have added a comprehensive "Related Work" section (lines 90-123) that discusses recent research in AI and LLMs for government-citizen communication, contextualizing our work within the field.

7. Provide more detailed information on the specific LLMs used and how RAG technology is implemented in the system.

Response: We have expanded our explanation of the system architecture (lines 354-371), detailing the use of GPT-3.5-turbo as our core LLM and explaining the implementation of RAG technology using FAISS for retrieval and Langchain for orchestration.

8. Elaborate on the characteristics of the dataset.

    Response: We have provided more detailed information about our dataset in the "Experiment Setup and Data Collection" section (lines 391-403), including the number of documents, variety of policy topics, and sources of the documents.

9. Provide more detailed descriptions of the metrics used to evaluate accuracy and comprehensibility.

    Response: We have expanded our discussion of evaluation metrics in the "Results and Performance Analysis" section (lines 433-448), detailing how accuracy was measured across different question types and regions.

10. Provide specific examples or case studies demonstrating how the system has improved engagement.

    Response: We have added a new "Application Scenarios" section (lines 521-555) that provides hypothetical scenarios illustrating how our system could enhance policy communication and citizen engagement in various contexts.

11. Describe a scenario where this system is implemented in a real-world government setting.

    Response: The new "Application Scenarios" section includes multiple hypothetical real-world scenarios, such as policy rollout, crisis communication, and complex policy explanation, demonstrating potential implementations of our system.

12. Compare your recommendations for using RAG technology and LLMs to existing methods for policy communication.

    Response: The new "Related Work" section (lines 90-123) provides comparisons between our approach and existing methods, highlighting the advantages of our RAG-based system.

13. Include a comparative analysis with previous studies.

    Response: While direct comparisons were challenging due to the novelty of our approach, we have acknowledged this limitation and provided context for our results in the "Results and Performance Analysis" section (lines 468-472).

14. Have the results been validated with independent datasets?

    Response: We have acknowledged the need for further validation in our "Conclusion and Discussion" section (lines 604-607), stating our intention to validate the system with independent datasets in future work.

15. Provide more detailed insights into potential biases introduced by these specific datasets.

    Response: We have addressed potential biases in our "Discussion and Limitations" section (lines 556-589), acknowledging the need for calibration to reflect diverse cultural, linguistic, and administrative contexts.

16. Provide specific scenarios where the proposed approach might face challenges in real-world applications.

    Response: The "Discussion and Limitations" section (lines 556-589) now includes a discussion of challenges our system might face in real-world applications, such as document structure and language complexity.

17. Describe a scenario where the early implementation of your system helped a government agency effectively communicate a new policy.

    Response: The new "Application Scenarios" section includes a hypothetical scenario (Scenario 1: Policy Rollout, lines 523-531) demonstrating how our system could aid in communicating a new environmental policy.

18. What steps would you recommend to ensure continuous performance and accuracy?

    Response: We have addressed this in our "Discussion and Limitations" section, recommending continuous refinement of NLP tools and standardization of policy documentation practices (lines 574-583).

19. Provide more details on the specific performance metrics used to evaluate the system.

    Response: We have expanded our discussion of performance metrics in the "Results and Performance Analysis" section (lines 433-448), detailing accuracy rates across different question types and regions.

20. Provide a scenario where the system's effectiveness might be challenged.

    Response: We have included two case studies of failed cases (lines 474-509) that illustrate scenarios where our system faced challenges, along with analysis of these failures.

21. Provide a detailed comparison of the limitations of traditional policy communication approaches and how your proposed model overcomes them.

    Response: The new "Related Work" section (lines 90-123) includes comparisons between traditional approaches and our model, highlighting how our system addresses limitations of existing methods.

22. What were the specific limitations encountered during the development and testing of the proposed system?

Response: We have discussed limitations and challenges in our "Discussion and Limitations" section (lines 556-589), including issues with document structure and language complexity.

Reviewer #2:

1. Lack of clear understanding of the article's genre.

   Response: We have restructured the paper to clearly present it as both an introduction to a novel tool and its experimental validation. The addition of the "Related Work" section (lines 90-123) and the restructured introduction provide a clearer context for our research.

2. Insufficient justification of the relevance and novelty of the research.

   Response: We have strengthened the justification of our research's relevance and novelty throughout the paper, particularly in the abstract, introduction, and the new "Related Work" section.

3. Modest description of the tool.

   Response: We have significantly expanded the description of our system in the "Our Approach to Enhancing Government-Citizen Communication" section (lines 214-304) and the "Details in the RAG Component in Our Approach" section (lines 304-388).

4. Insufficient integration of results into existing body of knowledge.

   Response: The new "Related Work" section (lines 90-123) and expanded "Discussion and Limitations" section (lines 556-589) now better integrate our results with existing literature and discuss the implications of our findings in the broader context of policy communication.

5. Absence of recent studies in the references.

   Response: We have updated our reference list to include more recent studies, particularly in the new "Related Work" section, ensuring our research is contextualized within the current literature.

We believe that these revisions have substantially improved our manuscript, addressing all the reviewers' comments and suggestions. The revised version now provides a clearer, more comprehensive, and well-contextualized presentation of our innovative approach to improving citizen-government interactions through AI. We thank the reviewers for their valuable input, which has helped us enhance the quality and impact of our work.

Best Regards,

Sheng Yun
